# The complete chloroplast genome sequence of *Litsea coreana* var*. lanuginosa* (Lauraceae): genome structure and phylogenetic analysis

**DOI:** 10.1080/23802359.2022.2116952

**Published:** 2022-09-08

**Authors:** Shanjun Tian, Yongpeng Li, Lili Xiao, Tengfei Ran

**Affiliations:** College of Agriculture, Guizhou University, Guiyang, China

**Keywords:** *Litsea coreana* var. *lanuginosa*, complete chloroplast genome, phylogeny

## Abstract

*Litsea coreana* var. *lanuginose* is a perennial, indeciduous, and broad-leaved tree used as an essential medicinal and edible plant. In addition, this species is well-known for its leaves are rich in aromatic oil. In this study, we firstly assembled and characterized the complete chloroplast genome of *L. coreana* var. *lanuginose* using Illumina pair-end sequencing and performed a phylogenetic analysis with other 13 species in Lauraceae. The results revealed that its chloroplast genome was 152,859 bp in total length with 39% of GC content, containing a pair of inverted repeats of 20,084 bp (IRA and IRB), separated by a large single-copy (LSC) region of 93,795 bp and a small single-copy (SSC) region of 18,896 bp. The plastid genome of *L. coreana* var. *lanuginose* encoded 125 genes, including 81 protein-coding genes, 36 transfer RNA (tRNA), and eight ribosomal RNA (rRNA) genes. The phylogenetic analysis suggested that *L. coreana* var. *lanuginose* was closely related to the clade of *Litsea monopetala*, *Litsea garrettii*, and *Litsea elongate* in Lauraceae family.

*Litsea coreana* var. *lanuginose* (Migo) Yang et P.H.Huang (in Act. Phytotax. Sin. 16 (4) 50. 1978), a variety of *Litsea coreana*, belonging to Lauraceae, is a small indeciduous tree with lanceolate leaf and widely distributes in the forests of central and southern China. *L.coreana* var. *lanuginose* contains medicinal compounds such as terpenoids, flavonoids, and alkaloids and its leaves are the raw material for making *Hawk-tea*, a traditional folk beverage and traditional Chinese medicine (Jia and Yuan 2019). However, there have been some debates over the systematic position of the genus *Litsea* and few studies on the *L. coreana* var. *lanuginose*, which significantly limit its development and utilization. Therefore, we assembled the completed chloroplast (cp) genome of *L. coreana* var. *lanuginosa* based on Illumina pair-end sequencing data (GenBank accession number: OL960662). The results of this study will provide meaningful information for the phylogeny of Lauraceae.

A cultivated *L. coreana* var*. lanuginosa* tree was collected from Zunyi city, Guiyang province, China (107°49’E, 27°77’N) and transplanted to the laboratory of Agriculture at Guizhou University for cultivation. No endangered or protected species were involved in the study, and no specific permissions were required for the sample. The voucher samples used in this study were deposited in the Herbarium of Forestry College at Guizhou University (Xingyong Cui, cuixy0520@163.com) under the accession number MT202108Lcl01. Total genomic DNA was extracted from 1 g of the fresh leaves using a modified CTAB method. The genomic sequencing was performed on the Illumina HiSeq Xten platform (Illumina, Shanghai, China) with a paired-end (PE150) strategy. All raw reads were filtered through the FastQC program to get clean reads with default parameters.

The obtained data were assembled by GetOrganelle (Jin et al. 2020) and then formed a circular cp genome using the Bandage program (Wick et al. 2015). The assembled structures and predictive genes of the cp genome were annotated with CPGAVAS2 (Shi et al. 2019) and compared with the cp sequence of *Neolitsea homilantha* as a reference, coupled with manual correction for start and stop codons of protein-coding genes. In addition, the tRNA genes were verified using the tRNAscan-SE program (Schattner et al. 2005). The SSR (Simple sequence repeat) and tandem repeats were validated by Misa and TFR programs. Phylogenetic analyses, including 38 Lauraceae and two Calycanthaceae species, were performed using complete cp genomes aligned by MAFFT v7.271 (Katoh and Standley 2013). All of the genomes were downloaded from NCBI GenBank. The maximum likelihood (ML) bootstrap analysis with 1000 replicates was performed using IQ-TREE v1.6.12 (Minh et al. 2020). TVM + F + I + G4 chosen according to BIC was selected as the best-fit model according to the built-in ModelFinder.

The complete cp genome of *L. coreana* var. *lanuginosa* was 152,859 base pairs (bp) with 39% GC content. The total plastid genome consisted of four distinct regions that are a large single-copy (LSC) region of 93,795 bp, a small single-copy (SSC) region of 18,896 bp, and a pair of inverted repeats (IR) regions of 20,084 bp. The quadripartite structure encodes 125 predictive genes in the cp genome, including 81 protein-coding genes, 36 transfer RNA (tRNA) genes, and eight ribosomal RNA (rRNA) genes. The phylogenetic analysis revealed that *L. coreana* var*. lanuginosa* and *L. coreana* var. *sinensis* formed a clade and were closely related to the clade of *L. szemaois* and *L. dilleniifolia* and the clade of *L. monopetala*, *L. garrettii*, *L. japonica*, and *L. elongate*. Then they formed a clade with other species of this genus adjacent to the genus *Lindera* (Figure 1).

**Figure 1.**
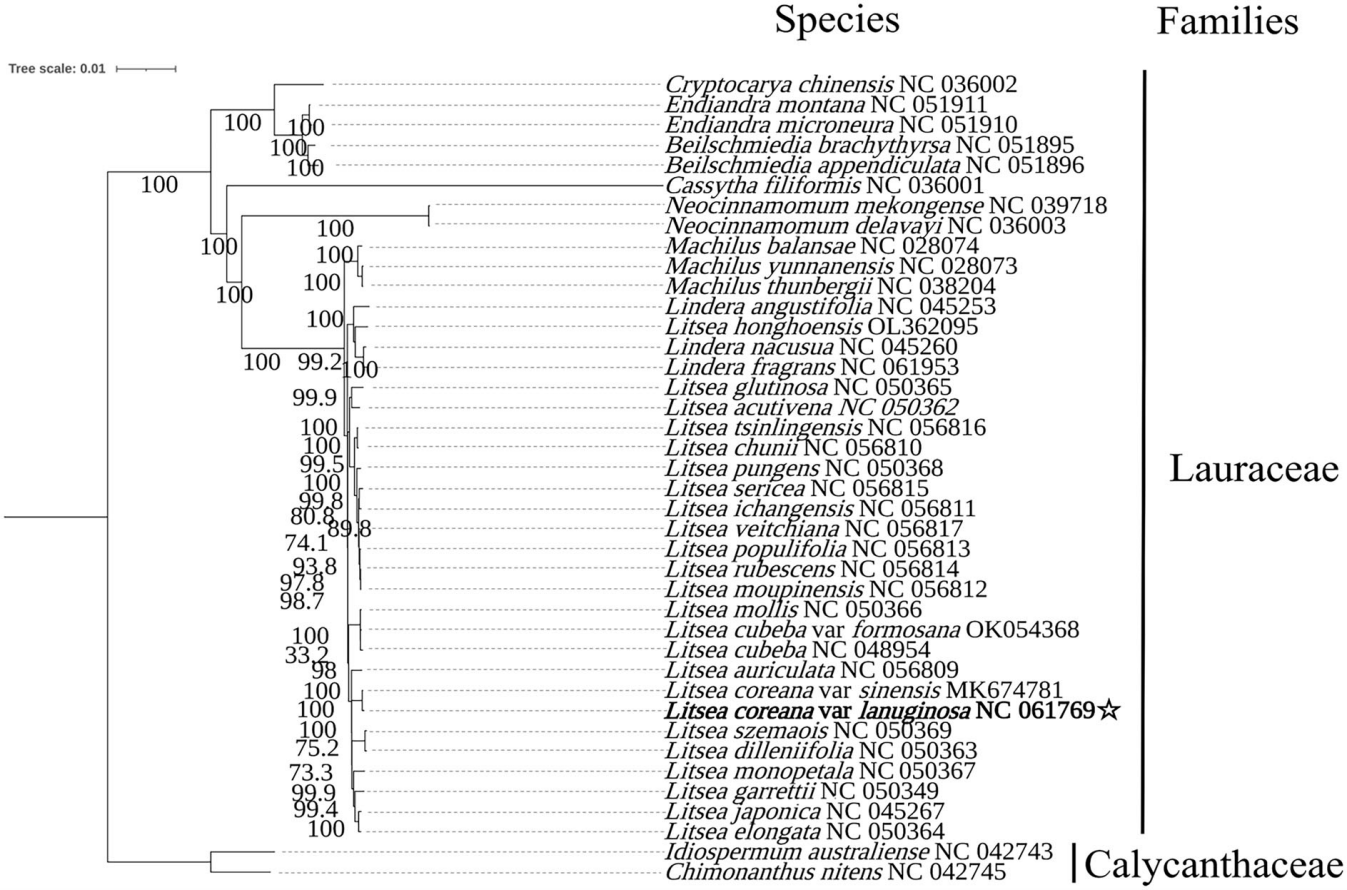
Maximum-likelihood phylogenetic tree of *L. coreana* var. *lanuginosa* based on complete cp genomes of 39 previously reported species. All the sequences were downloaded from NCBI GenBank. Numbers on the nodes are bootstrap values from 1000 replicates.

## Author contributions

ST conceived the project and designed the study; YL, LX, and TR performed the sampling and experiments; ST performed the data analysis and wrote the manuscript; ST edited the manuscript; and all authors read and approved the final manuscript.

## Data Availability

The complete chloroplast genome has been deposited in GenBank (accession number NC061769). The assembled individual was linked with no. SAMN25010760, Project ID: PRJNA797553, and SRA no. SRR17731627.
